# Focused ultrasound of the pleural cavities and the pericardium by nurses after cardiac surgery

**DOI:** 10.3109/14017431.2015.1009383

**Published:** 2015-02-09

**Authors:** Torbj⊘rn Graven, Alexander Wahba, Anne Marie Hammer, Ove Sagen, Øystein Olsen, Kyrre Skjetne, Jens Olaf Kleinau, Havard Dalen

**Affiliations:** 1 Department of Medicine, Levanger Hospital, Nord-Tr⊘ndelag Health Trust, Levanger, Norway; 2 Department of Cardiothoracic Surgery, Trondheim University Hospital/St Olavs Hospital, Trondheim, Norway; 3 MI Lab and Department of circulation and medical imaging, Norwegian University of Science and Technology, Norway; 4 Department of Radiology, Levanger Hospital, Nord-Tr⊘ndelag Health Trust, Norway

**Keywords:** echocardiography, pocket-size, PSID, postoperative, training

## Abstract

*Objectives.* We aimed to study the feasibility and reliability of focused ultrasound (US) examinations to quantify pericardial (PE)- and pleural effusion (PLE) by a pocket-size imaging device (PSID) performed by nurses in patients early after cardiac surgery. *Design.* After a 3-month training period, with cardiologists as supervisors, two nurses examined 59 patients (20 women) with US using a PSID at a median of 5 days after cardiac surgery. The amount of PE and PLE was classified in four categories by US (both) and chest x-ray (PLE only). Echocardiography, including US of the pleural cavities, by experienced cardiologists was used as reference. *Results.* Focused US by the nurses was more sensitive than x-ray to detect PLE. The correlations of the quantification of PE and PLE by the nurses and reference was *r* (95% confidence interval) 0.76 (0.46–0.89) and 0.81 (0.73–0.89), both *p* < 0.001. PE and PLE were drained in one and six (eight cavities) patients, all classified as large amount by the nurses. *Conclusions.* Cardiac nurses were able to obtain reliable measurements and quantification of both PE and PLE bedside by focused US and outperform the commonly used chest x-ray regarding PLE after cardiac surgery.

## Introduction

Pericardial (PE)- and pleural effusion (PLE) are common complications of cardiac surgery and may cause increased morbidity, prolonged hospitalization or hospital readmission (1,2). Pericardial tamponade may even be life threatening when it leads to haemodynamic compromise (3). In clinical practice, the detection and follow-up of PE and PLE are frequently done by physical examination and chest x-ray. The diagnostic accuracy of physical examination in this context is clearly inferior and standard chest x-ray has been shown to be inaccurate in detecting and quantifying effusions (4–7). Both thoracic computed tomography (CT) and magnetic resonance (MR) imaging are widely accepted diagnostic methods for the detection of PLE and PE, but have the dis-advantages of being resource-intensive, as well as the use of intravenously administered contrast (8).

Ultrasound (US) has been proven to be a sensitive and accurate tool to detect and quantitate PE and PLE (6,7). It can be performed rapidly and bedside. Hand-carried ultrasound devices have been shown to be useful in detecting both PE and PLE after cardiac surgery (9,10). The development of low cost, pocket-size imaging devices (PSID) has promoted the widespread use of such devices in different medical specialties and clinical scenarios (11–14). Thus, it may be hypothesized that routinely implementing focused US examinations to patients in the postoperative period after surgery may improve follow-up as PE and PLE can be revealed and quantified prior to symptoms.

We aimed to study the feasibility and reliability of focused US of the pericardium and the pleural cavities by PSID performed by nurses (focused US) in patients in the early phase after cardiac surgery and study the sensitivity and accuracy to detect clinically significant PLE compared with chest x-ray.

## Methods

### Study population

In this prospective single-centre observational study patients from Nord-Tr⊘ndelag County in Norway requiring cardiac surgery were included. This corresponds to 15–20% of patients undergoing cardiac surgery at the regional Trondheim University Hospital, Trondheim, Norway. By regional medical protocol, all the patients from Nord-Tr⊘ndelag County are transferred from the Trondheim University Hospital to the non-university Levanger Hospital in the early postoperative period for recovery, mostly on the third postoperative day. Inclusion criteria were only that patients had undergone cardiac surgery and were transferred to the non-university hospital. Patients were available for inclusion in the period of April 29th 2013 to December 23rd 2013.

The only exclusion criterion was unwillingness or inability to provide informed consent. Before entering the study, all patients provided written informed consent to undergo focused US performed by nurses to assess PE and PLE followed by reference examination. The study was registered at ClinicalTrials.gov, ID: NCT01847859 and approved by the Regional Committee for Medical and Health Research Ethics and conducted according to the 2nd Declaration of Helsinki.

### Pre study training of the nurses

Before starting the study, two nurses specialized in cardiology underwent a 3-month training period of focused US with cardiologists as supervisors. They had no previous experience with diagnostic US. All education were given bedside with hands-on training, as well as the nurses practiced on their own on patients who were undergoing echocardiography with suspected PE or PLE. During the training period the nurses performed 62 and 58 supervised focused US examinations, respectively. The focused US examination aimed to assess the pericardial and pleural cavities in patients in the early postoperative phase after cardiac surgery with respect to detect and quantify the amount of PE and PLE.

### Focused pocket-size ultrasound examination by nurses

The US examinations by the two nurses were performed with the PSID Vscan (version 1.2; GE Ultrasound AS, Horten, Norway). The device weighs 390 g, image sector is 75°, bandwidth is automatically adjusted (1.7–3.8 MHz) and both grey scale and colour Doppler modes are available in real time. Patient identification was possible using the voice recording. All images and recorded loops were saved on the PSID's micro-SD card and later transferred to a computer by commercial software (Gateway; GE Vingmed Ultrasound).

**Figure 1.**
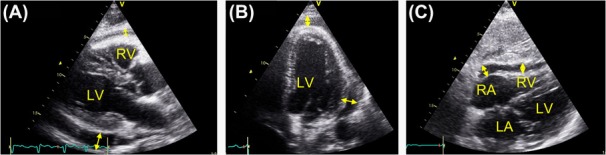
Echocardiography with pericardial effusion. A: parasternal long axis view. B: apical four-chamber view. C: subcostal view. *Double arrows* indicate the measure points of pericardial effusion. LV, left ventricle; RV, right ventricle; LA, left atrium; RA, right atrium.

The assessment of the pericardial cavity for PE was done by two-dimensional (2D) views. With the patient placed in a left lateral decubitus position, parasternal long and short axis and apical four-chamber views were obtained, and with the patient in a supine position a subcostal 2D four-chamber view was obtained. PE was defined to be present when a hypoechoic space was visualized between the epicardium and the pericardium. As there is no standardized measurement for the quantification of PE by 2D echocardiography, the PE, if present, was quantified as the average of measurements of the largest end-diastolic distance between epicardium and pericardium at four points (alongside the left ventricle, at the apex, the right ventricle and the right atrium) (Figure 1). The amount of PE was classified as 1) not present, 2) insignificant if the maximum dimension of each measurement was < 5 mm, 3) moderate (5–14 mm) and 4) large if maximum dimension of at least one measurement was ≥ 15 mm.

**Figure 2.**
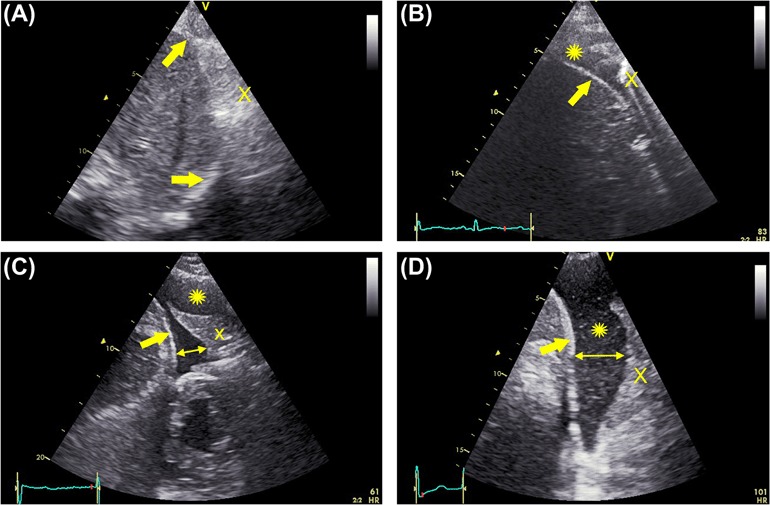
Chest ultrasound with examples of pleural effusion. A: absent, B: small (only in the costophrenic angle), C: moderate, D: large. *Double arrows* show the measurement of pleural effusion. *Thick arrows* indicate the diaphragm. X: lung (in B air-filled, in C and D consolidated). ∗pleural effusion.

After completing the focused echocardiography, thoracic ultrasonography was performed with the same device with the patient in sitting position. With the transducer placed in the intercostal space, the liver and spleen were used as landmarks to identify the diaphragm of the right and left hemithoraces, respectively. During quiet breathing, US scanning of the posterior chest was performed along the paravertebral, scapular, posterior and medial axillary lines, continuously focusing on the diaphragm as a landmark. The air-filled lung surface results in a bright line and distal shadows, indicating the absence of PLE (Figure 2A). The presence of PLE was diagnosed by the appearance of a hypoechoic space between the diaphragm and the air-filled or consolidated lung surface (Figure 2B). If the PLE was located in the costodiaphragmatic angle only, this was assessed semi quantitatively and classified as insignificant (Figure 2C). By larger effusions the dimension between the diaphragm and the lung surface was measured in the middle, between the transducer and the mediastinum. If a consolidated lung, yielding a tissue pattern, bulged into the effusion, the extent of the effusion was measured just medially to the protruding edge of the lower lung lobe (Figure 2D). Dimensions were measured in real time on the PSID. For each pleural cavity, the amount of PLE was classified as: 1) not present, 2) insignificant (costodiaphragmatic angle only), 3) moderate if the PLE separated the diaphragm and the lung with a maximum distance between these two organs < 30 mm and 4) large if this maximum distance was ≥ 30 mm.

### Comparison with other imaging methods

For the study purpose all patients underwent reference imaging. The reference echocardiography was performed by one of the four cardiologists, all experienced in echocardiography and ultrasound examinations of the pleural cavities (each > 2000 examinations), in a random matter, and blinded to the results of the US examination by the nurse. A Vivid E9 scanner (BT12; GE Ultrasound, Horten, Norway) was used. The dimension of PE and PLE was measured as described for pocket-size ultrasound. Echocardiography examination was performed with the patient placed in a left lateral supine position. The image quality of echocardiography was scored from 1 (poor) to 3 (good) based on visual assessment by the operator and one additional cardiologist experienced in echocardiography. Ejection fraction and left ventricular volumes were calculated based on end-diastolic and end-systolic tracings in the 4-chamber and 2-chamber views and left ventricular dimension was measured in motion-mode recordings of the parasternal long axis. Mitral inflow was assessed by pulsed wave Doppler with the sample volume at the tip of the mitral leaflets. These measurements are used for the description of the study population only.

One experienced radiologist at the non-university hospital blinded to clinical data and the results of the US examinations interpreted all the chest x-rays. PLE was classified as: 1) not present, 2) small if present in costodiaphragmatic angle only, 3) moderate if present in the lower hemithorax, but not exceeding the fourth rib, and 4) large if exceeding the fourth rib.

### Statistics

As the different echocardiographic and anthropometric measures partly were skewed, the basic characteristics are presented as mean ± standard deviation (SD) and range. For comparison of continuous variables, Pearson's rho (*r*) and Bland–Altman statistics were used. PLE was given the value “0” if not present and the value “5 mm” if present in the costodiaphragmatic angle only. Spearman's rank correlation was used for testing of the correlations between non-parametric data. Data are presented as *r* (95% confidence interval (CI)) with the 95% CI computed using bootstrapping. The ability to detect the specific amounts of PLE was tested by assessing the sensitivity, specificity, positive and negative predictive values and receiver operator curves (ROC) using the classification by the cardiologist as the reference. For the comparison of focused US by the nurses and the chest x-ray with respect to detect specific amounts of PLE, the area under the ROC curve and the respective 95% CI was used. Comparison of proportions was analysed by chi-square statistics. A two-sided *p* < 0.05 was considered statistical significant. All the statistical analyses were performed using SPSS for Windows (version 21, SPSS, Inc., Chicago, IL, USA).

## Results

### Study population

Table I summarizes the basic characteristics of the study population. Of 59 screened patients all were included in the study. Of this population 30 underwent solely coronary artery bypass graft (CABG) surgery, 13 patients solely valve surgery, 12 patients CABG plus valve surgery, 2 patients solely ascending aortic graft surgery and 2 patients ascending aortic graft plus (aortic) valve surgery. All the patients were examined once by focused US by the nurses at first day-time occasion (7/7 days) median (range) 5.0 (3.2–31) days after surgery.

**Table I.** Basic characteristics of the 59 study participants.

	Median (range)
Age, years	67 (35–86)
Females, n (%)	20 (34)
Body mass index, kg/m^2^	27.6 (19.0–44.5)
Procedures CABG/Valve/Aorta (% pts)	71/42/7
Sinus rhythm/AFIB (% pts)	86/12
Time from surgery to focused ultrasound by the nurses, days	5.0 (3.2–31)
Time consumption focused ultrasound by the nurses, minutes	12.6 (7.0–19.0)
Time from ultrasound by the nurses to reference, hours	3.4 (0.3–27)
Time from chest x-ray to ultrasound by the nurses, hours	25 (0–73)
Image quality (Scale poor = 1 to good = 3), mean (SD)	1.9 (0.7)
Left ventricular end-diastolic volume, ml	113 (52–233)
Ejection fraction, %	53 (30–75)

Data are presented as median (range) if not specified elsewhere.

AFIB, atrial fibrillation; CABG, coronary artery bypass graft; n, number; pts, patients; SD, standard deviation

### Pocket-size ultrasound performed by nurses to assess pericardial- and pleural effusion, and in addition, the use of chest x-ray for detection of pleural effusion

The nurses were able to assess and evaluate both the pleural cavities and the pericardial cavity with respect to PLE and PE in 59 of the 59 participants (Table II). Similarly, all patients were completely assessed regarding both PLE and PE by the reference examination. The nurses identified all patients who had to undergo drainage of PE or PLE due to large amount of PE and PLE. Image quality of the reference echocardiography was mean (SD) 1.9 (0.7). Time consumption for the US examination by nurses was median (range) 13 (7–19) minutes and the time interval between the US examinations by the nurses and the reference echocardiography were mean (SD) 3 (5) hours. The time interval between chest x-ray and reference echocardiography was 27 (25) hours, respectively. In 47 (80%) patients, an upright posteroanterior and lateral chest x-ray was performed. In the 12 remaining patients, the beam direction was anteroposterior, only.

**Table II.** Feasibility of focused pocket-size imaging of the pericardium and the pleural cavities performed by nurses and the distribution of pathology in the study population.

	Pericardium	Pleural cavities
Feasibility of ultrasound by the nurses, *N* (%)	59 (100%)	118 (100%)
Number of cavities with significant amount of effusion, *N* (%)	36 (61%)	95 (81%)
Measures of effusion (mm), mean ± SD (range)^∗^	5 ± 3 (1–18)	29 ± 14 (8–60)

∗ The mean, SD and range of effusion among the 36 pericardial- and 95 pleural cavities with significant amount of effusion.

N, number; SD, standard deviation

Tables II and III show the high prevalence and the distribution of the PE and PLE. The type of surgery had no significant effect on the presence of at least moderate PE or PLE, but two (of two) patients in need of therapy for PE underwent aortic valve surgery.

**Table III.** Correlations of focused ultrasound by the nurses and chest x-ray with reference.

	Number of cavities with pathology^∗^	*r* (95% CI)	p-value
Pericardial effusion (PSID_nurses_ vs reference)	34	0.76 (0.46–0.89)	< 0.001
Pleural effusion (PSID_nurses_ vs reference)	109	0.81 (0.73–0.89)	< 0.001
Pleural effusion (chest x-ray vs reference)	109	0.21 (0.04–0.37)	0.03

∗ The correlations of pericardial- and pleural effusions were tested in 59 and 118 cavities, respectively. For pericardial effusion pathology is classified as present if maximum dimension in at least one measurements was at least 5 mm. Pleural effusion pathological if present.

CI, confidence interval; PSID_nurses_, focused pocket-size ultrasound by the nurses

**Figure 3.**
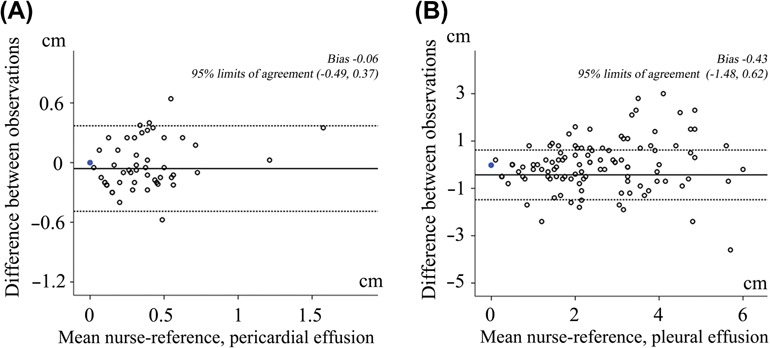
Bland–Altman plots of the difference of measurements of pericardial and pleural effusion by the nurses and reference plotted against the means of the measurements.

**Figure 4.**
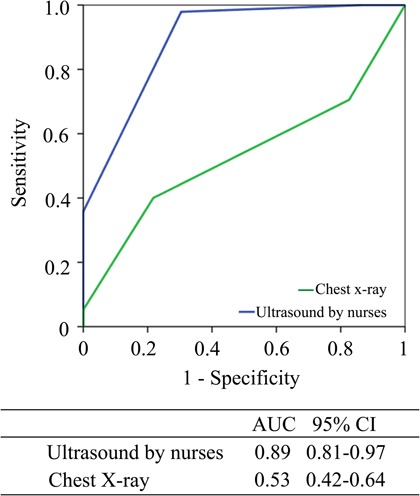
Receiver operating characteristics curve of pocket-size ultrasound by nurses and chest x-ray with respect to detect at least moderate pleural effusion. Reference is high-end examination by cardiologist. AUC, area under curve; CI, confidence interval.

The correlation of the quantification of PE and PLE performed by the nurses and the reference method was high with *r* (95% CI) 0.76 (0.46–0.89) and 0.81 (0.73–0.89), both *p* < 0.001, respectively (Table III). There was no significant difference between the two nurses compared to reference regarding the measurements of PE and PLE, both *p* ≥ 0.29. The corresponding correlation of chest x-ray with reference was low with *r* (95% CI) 0.21 (0.04–0.37), *p* = 0.03. The sensitivity and specificity to detect at least moderate PE by US performed by the nurses was 91% and 56%, respectively. In 11 cases, nurses classified the amount of PE as moderate while the cardiologist classified the amount as less (< 5 mm). For PLE, the corresponding sensitivity and specificity was 98% and 70% for focused ultrasound by the nurses and 40% and 78% by chest x-ray, respectively (Table IV). The low sensitivity of chest x-ray to detect PLE was illustrated in further analyses. Detection of PLE exceeding the costodiaphragmatic angle by chest x-ray had only a sensitivity of 53% to detect large amount of pleural effusion classified by reference. In two (3%) patients PE was quantified as large, and both were correctly identified by the nurses. The Bland–Altman plots illustrate no significant reduction in the accuracy of the measurements of PE and PLE performed by nurses with larger amount of PE or PLE (Figure 3). In Figure 4, the superiority of focused US compared with chest x-ray for the assessment of PLE, using high-end echocardiography by the cardiologists as the reference, is shown. Whether the chest x-ray was performed by standard beam directions or anteroposterior beam direction only did not alter the results. The area under the ROC curve for chest x-ray performed with standard versus anteroposterior beam direction was 0.56 (0.26–0.84) versus 0.55 (0.42–0.67), respectively.

**Table IV.** Sensitivity, specificity, positive and negative predictive value of nurse performed focused pocket-size imaging by nurses and chest x-ray to detect at least moderate pathology in patients after cardiac surgery.

	N total (% patho)	Sensitivity (%)	Specificity (%)	PPV (%)	NPV (%)
Pericardial effusion (PSID_nurses_)	59 (61%)	91	56	74	82
Pleural effusion (PSID_nurses_)	118 (81%)	98	70	93	89
Pleural effusion (chest x-ray)	118 (81%)	40	78	88	24

N, numbers; NPV, negative predictive value; PPV, positive predictive value; PSID_nurses_, focused pocket-size ultrasound by the nurses

## Discussion

The present study shows that nurses specialized in cardiology were able to perform focused US to detect and quantify PE and PLE with a PSID with a high feasibility of 100% after a relatively short period of training. Moreover, the nurses were able to reliably detect and quantify both PE and PLE despite the fact that the image quality in this early postoperative period after cardiac surgery was suboptimal. Compared to chest x-ray focused US by the nurses was significantly superior with respect to the detection of PLE. This allows for providing essential information of frequent complications as PE and PLE following cardiac surgery to the physicians caring for these patients at an early stage. The frequent finding of PE and PLE in the early postoperative phase after cardiac surgery is in line with other studies (9,15). In total seven patients (nine cavities) were in need of drainage of PE and PLE.

Furthermore, it may be difficult to detect small, insignificant effusions, particularly with respect to PE. The use of the low cost, miniaturized PSID pose a challenge in this respect, as the suboptimal environment when performing the US examinations bedside (16). With respect to the comparison of US with chest x-ray, both different classifications and the time difference between the examinations may influence the results. Chest x-ray was less sensitive to detect all levels of PLE compared to focused US, and even by including all pleural cavities with more than moderate or large amount of PLE by chest x-ray the sensitivity with respect to detect large amount was only 53%. The findings of this study illustrate the feasibility that nurses in a cardiac or postoperative ward can be trained in focused US to detect complications following cardiac surgery at an early stage. It is important to state that this approach is based on the responsibility of the physician for the follow-up and the application of this information. However, in our opinion an approach where nurses or other health-care personnel are educated in the use of focused US may significantly contribute to improve the caregiving physicians' decision-making.

In the early postoperative phase after cardiac surgery, the image quality of echocardiography is frequently suboptimal (17,18). In addition to image quality the lack of standardized measures regarding the assessment of PE and PLE is challenging when comparing separate examinations. This may partly explain the suboptimal sensitivity and specificity in our study. Nevertheless, all patients with clinically significant PE and PLE requiring intervention whether with medical treatment or drainage were identified by the nurses as large amounts. Two patients needed treatment for large amount of PE (one received treatment with colchicine and one underwent pericardial drainage). However, in this relatively small study the type of surgery was not significantly associated with the detection of at least moderate amount of PE and PLE. Mostly the PE and PLE disappear without specific therapy, as in our study (10,19).

The majority of PE and PLE tends to be asymptomatic or the symptoms are non-specific, even for cardiac tamponade (2,20). The risk of misjudging the situation may be substantial as physical examination and chest x-ray may underestimate PE and PLE (4–7,21). Missing a timely diagnosis of PE or PLE may lead to inappropriate treatment and have deleterious consequenses (22). US can provide rapid and accurate diagnosis of both PE and PLE as shown by this study and others (9,10,16,23). The easy access of ultrasonography, particularly PSID, may facilitate a correct and timely diagnosis and thus contribute to prevent the development of life threatening conditions such as cardiac tamponade or respiratory failure (22,24). Chest x-ray is not sensitive enough to be used in everyday clinical practice, and even though the use of low dose radiation CT could perform well, it is more resource-intensive and time consuming than PSID.

Point-of-care ultrasonography is defined as ultrasonography brought to the patient (25). The development of PSID has made focused US easily available. It has gained widespread use and several studies have shown high feasibility and reliability in various clinical settings used by experts as well as by novices (16,23). Appropriate training specifically tailored to the information which may be requested (focused US) is essential and may facilitate the use among different medical professions (11,12,14). In our experience the described approach of a 3-month period of supervised training including 60 focused US examinations of the pleural and pericardial cavities seems adequate. Being able to perform focused US to detect PE and PLE has the potential to minimize the time window without US competence available and might provide the nurses with information which would lead to a timely call for a doctor in situations where PE or PLE could be the cause of clinical deterioration.

The access to a cardiologist or other health-care professionals being able to perform echocardiography and chest US on a 24/7 basis may be an ideal alternative to the method used in this study. How the use of focused US in the early postoperative phase after cardiac surgery could be the implemented in daily clinic will vary between different hospitals, but this study shows that health-care personnel, even without former experience in US, can be trained to collect important information by focused US for the caregiving physicians.

## Conclusion

After tailored training, nurses were able to perform focused US with PSID and reliable detect and quantify PE and PLE in patients in the early phase after cardiac surgery. The US examinations performed by the nurses were superior to chest x-ray to detect and quantify PLE. Implementing focused US performed by nurses may allow for making essential information available for the caregiving physician and contribute to a safer and better postoperative care of these patients.

## Notes

***Declaration of interest:*** H Dalen holds position at the Medical Imaging Laboratory, NTNU, a Centre of Research-based Innovation that is funded by the Research Council of Norway and industry. One of the industry partners is GE Vingmed Ultrasound. The Centre has a total budget of app. 124 million NOK for the 8-year period 2007–2014, and the contribution from GE Vingmed Ultrasound to this budget is approximately 7 million NOK (< 6%).
